# *rac*-Methyl 2-(2-formyl-4-nitro­phen­oxy)hexa­noate

**DOI:** 10.1107/S1600536812015462

**Published:** 2012-04-18

**Authors:** Jun-Song Song, De-Cai Wang, Xue-Jun He, Jiang-Kai Qiu, Ping-Kai Ou-yang

**Affiliations:** a State Key Laboratory of Materials-Oriented Chemical Engineering, School of Pharmaceutical Sciences, Nanjing University of Technology, Xinmofan Road No. 5 Nanjing, Nanjing 210009, People’s Republic of China

## Abstract

In the racemic title compound, C_14_H_17_NO_6_, the plane of the ester group of the methyl hexa­noate side chain makes a dihedral angle of 80.0 (2)° with the benzene ring, while the nitro group is approximately coplanar with the benzene ring [dihedral angle = 10.3 (2)°]. In the crystal, mol­ecules form weak aromatic C—H⋯O_nitro_ hydrogen-bonding inter­actions, giving inversion dimers [graph set *R*_2_^2^(8)].

## Related literature

For applications of the title compound, see: Dale & White (2007 ▶). For graph-set analysis, see: Etter *et al.* (1990 ▶)
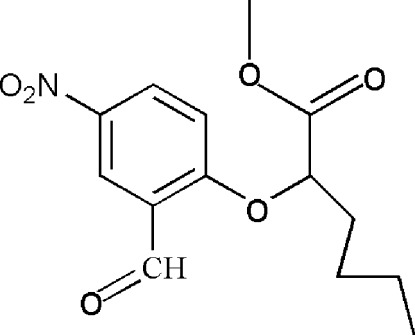


## Experimental

### 

#### Crystal data

C_14_H_17_NO_6_
*M* *_r_* = 295.29
Monoclinic, 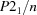

*a* = 14.918 (3) Å
*b* = 4.922 (1) Å
*c* = 20.928 (4) Å
β = 103.26 (3)°
*V* = 1495.7 (5) Å^3^
*Z* = 4
Mo *K*α radiation
μ = 0.10 mm^−1^
*T* = 293 K
0.20 × 0.10 × 0.10 mm

#### Data collection

Enraf–Nonius CAD-4 four-circle diffractometer
Absorption correction: ψ scan (North *et al.*, 1968 ▶) *T*_min_ = 0.980, *T*_max_ = 0.990
2722 measured reflections
2722 independent reflections
1228 reflections with *I* > 2σ(*I*)
3 standard reflections every 200 reflections intensity decay: 1%

#### Refinement

*R*[*F* ^2^ > 2σ(*F* ^2^)] = 0.076
*wR*(*F* ^2^) = 0.172
*S* = 1.00
2722 reflections
172 parameters
H-atom parameters constrained
Δρ_max_ = 0.26 e Å^−3^
Δρ_min_ = −0.22 e Å^−3^

### 

Data collection: *CAD-4 EXPRESS* (Enraf–Nonius, 1994 ▶); cell refinement: *CAD-4 EXPRESS*; data reduction: *XCAD4* (Harms & Wocadlo, 1995 ▶); program(s) used to solve structure: *SHELXS97* (Sheldrick, 2008 ▶); program(s) used to refine structure: *SHELXL97* (Sheldrick, 2008 ▶); molecular graphics: *SHELXTL* (Sheldrick, 2008 ▶); software used to prepare material for publication: *SHELXTL*.

## Supplementary Material

Crystal structure: contains datablock(s) I, global. DOI: 10.1107/S1600536812015462/zs2196sup1.cif

Structure factors: contains datablock(s) I. DOI: 10.1107/S1600536812015462/zs2196Isup2.hkl

Supplementary material file. DOI: 10.1107/S1600536812015462/zs2196Isup3.cml

Additional supplementary materials:  crystallographic information; 3D view; checkCIF report

## Figures and Tables

**Table 1:** Hydrogen-bond geometry (Å, °)

*D*—H⋯*A*	*D*—H	H⋯*A*	*D*⋯*A*	*D*—H⋯*A*
C2—H2*A*⋯O1^i^	0.93	2.52	3.442 (6)	169

Symmetry code: (i) 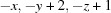.

**Fig. 1.**
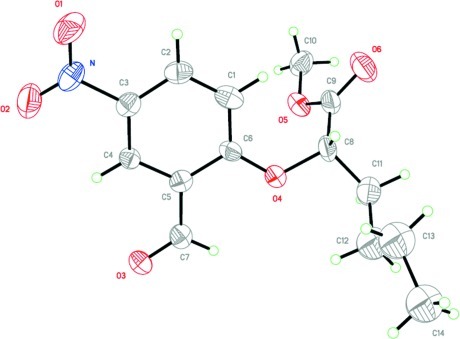
The structure of the title compound, showing the atom numbering scheme. Non-H atoms are shown as 30% probability displacement ellipsoids.

## supplementary crystallographic information

### Comment

The title compound, C_14_H_17_NO_6_ is a good organic intermediate for the synthesis of the drug dronedarone, an important drug used to treat cardiac arrhythmia (Dale & White, 2007), and its crystal structure is reported herein.

In the title compound (Fig. 1), both the nitro group and the aldehyde group are approximately coplanar with the benzene ring, as shown by the torsion angles O1—N—C3—C4 [170.9 (4)°] and C6—C5—C7—O3 [177.6 (4)°]. The plane of the ester group of the methyl hexanoate side chain makes a dihedral angle of 80.0 (2)° with the benzene ring. In the crystal, the molecules are linked by weak intermolecular aromatic C2—H···O1_nitro_ hydrogen-bonding interactions (Table 1), giving centrosymmetric cyclic dimers [graph set *R*^2^_2_(8) (Etter *et al.*, 1990)]. Also present are intramolecular interactions between the aldehyde and methylene C—H groups and the ether O-atom.

### Experimental

A mixture of 5-nitrosalicylaldehyde (0.2 mol, 33.4 g), methyl 2-bromohexanoate (2-bromhexine acid methyl ester) (0.2 mol, 41.8g) and anhydrous potassium carbonate (0.2 mol, 27.6g) in DMF (400 ml) was reacted for 3.5h at 365-367 K. After the completion of the reaction, the precipitate was filtered and washed and the product (0.1 g) was crystallized from 15 ml of CH_3_OH at room temperature to give colorless crystals from which a specimen was selected for X-ray data collection.

### Refinement

All H atoms were placed in calculated positions and treated as riding, with C—H = 0.93, 0.98, 0.97 and 0.96 Å for CH(aromatic), C—H(aliphatic), CH, CH_2_ and CH_3_ H atoms, respectively and with *U*_iso_(H) = k × *U*_eq_(C), where k = 1.5 for CH_3_ H-atoms and k = 1.2 for all other H-atoms.

### Crystal data

**Table:**

C_14_H_17_NO_6_	*F*(000) = 624
*M**_r_* = 295.29	*D*_x_ = 1.311 Mg m^−^^3^
Monoclinic, *P*2_1_/*n*	Mo *K*α radiation, λ = 0.71073 Å
Hall symbol: -P 2yn	Cell parameters from 25 reflections
*a* = 14.918 (3) Å	θ = 9–13°
*b* = 4.922 (1) Å	µ = 0.10 mm^−^^1^
*c* = 20.928 (4) Å	*T* = 293 K
β = 103.26 (3)°	Block, colourless
*V* = 1495.7 (5) Å^3^	0.20 × 0.10 × 0.10 mm
*Z* = 4	

### Data collection

**Table:**

Enraf–Nonius CAD-4 four-circle diffractometer	1228 reflections with *I* > 2σ(*I*)
Radiation source: fine-focus sealed tube	*R*_int_ = 0.000
Graphite monochromator	θ_max_ = 25.4°, θ_min_ = 1.5°
ω–2θ scans	*h* = −17→17
Absorption correction: ψ scan (North et al., 1968)	*k* = 0→5
*T*_min_ = 0.980, *T*_max_ = 0.990	*l* = 0→25
2722 measured reflections	3 standard reflections every 200 reflections
2722 independent reflections	intensity decay: 1%

### Refinement

**Table:**

Refinement on *F*^2^	Primary atom site location: structure-invariant direct methods
Least-squares matrix: full	Secondary atom site location: difference Fourier map
*R*[*F*^2^ > 2σ(*F*^2^)] = 0.076	Hydrogen site location: inferred from neighbouring sites
*wR*(*F*^2^) = 0.172	H-atom parameters constrained
*S* = 1.00	*w* = 1/[σ^2^(*F*_o_^2^) + (0.050*P*)^2^] where *P* = (*F*_o_^2^ + 2*F*_c_^2^)/3
2722 reflections	(Δ/σ)_max_ < 0.001
172 parameters	Δρ_max_ = 0.26 e Å^−^^3^
0 restraints	Δρ_min_ = −0.22 e Å^−^^3^

### Special details

**Table:**

Geometry. All esds (except the esd in the dihedral angle between two l.s. planes) are estimated using the full covariance matrix. The cell esds are taken into account individually in the estimation of esds in distances, angles and torsion angles; correlations between esds in cell parameters are only used when they are defined by crystal symmetry. An approximate (isotropic) treatment of cell esds is used for estimating esds involving l.s. planes.
Refinement. Refinement of F^2^ against ALL reflections. The weighted R-factor wR and goodness of fit S are based on F^2^, conventional R-factors R are based on F, with F set to zero for negative F^2^. The threshold expression of F^2^ > 2sigma(F^2^) is used only for calculating R-factors(gt) etc. and is not relevant to the choice of reflections for refinement. R-factors based on F^2^ are statistically about twice as large as those based on F, and R- factors based on ALL data will be even larger.

### Fractional atomic coordinates and isotropic or equivalent isotropic displacement parameters (Å^2^)

**Table:**

	*x*	*y*	*z*	*U*_iso_*/*U*_eq_	
N	−0.0326 (2)	0.6463 (8)	0.59117 (19)	0.0672 (11)	
C1	0.2020 (3)	0.5437 (8)	0.56249 (18)	0.0592 (11)	
H1A	0.2389	0.6148	0.5363	0.071*	
O1	−0.0535 (2)	0.8534 (7)	0.55953 (18)	0.0926 (11)	
C2	0.1162 (3)	0.6501 (9)	0.55803 (19)	0.0604 (11)	
H2A	0.0946	0.7942	0.5300	0.072*	
O2	−0.0834 (2)	0.5218 (8)	0.61742 (19)	0.1013 (13)	
C3	0.0617 (3)	0.5338 (9)	0.59740 (18)	0.0544 (10)	
O3	0.16665 (19)	−0.0836 (7)	0.72916 (13)	0.0747 (10)	
O4	0.31794 (16)	0.2096 (6)	0.61157 (12)	0.0598 (8)	
C4	0.0913 (2)	0.3232 (7)	0.64028 (16)	0.0437 (9)	
H4A	0.0533	0.2496	0.6654	0.052*	
C5	0.1794 (2)	0.2243 (8)	0.64485 (17)	0.0496 (9)	
O5	0.2966 (2)	0.0517 (6)	0.48813 (13)	0.0675 (9)	
C6	0.2353 (3)	0.3330 (8)	0.60504 (17)	0.0529 (10)	
O6	0.3773 (2)	0.3910 (8)	0.46304 (16)	0.0990 (12)	
C7	0.2119 (3)	0.0063 (8)	0.69327 (17)	0.0549 (10)	
H7A	0.2704	−0.0651	0.6961	0.066*	
C8	0.3791 (3)	0.3212 (9)	0.5738 (2)	0.0645 (12)	
H8A	0.3839	0.5183	0.5803	0.077*	
C9	0.3496 (3)	0.2609 (11)	0.5030 (2)	0.0663 (12)	
C10	0.2656 (3)	−0.0163 (11)	0.41826 (19)	0.0806 (15)	
H10A	0.2268	−0.1741	0.4135	0.121*	
H10B	0.3180	−0.0529	0.4003	0.121*	
H10C	0.2315	0.1337	0.3953	0.121*	
C11	0.4715 (3)	0.1900 (12)	0.6028 (2)	0.0907 (17)	
H11A	0.4652	−0.0034	0.5941	0.109*	
H11B	0.5153	0.2588	0.5790	0.109*	
C12	0.5116 (4)	0.2243 (12)	0.6721 (3)	0.107	
H12A	0.4667	0.1703	0.6967	0.128*	
H12B	0.5642	0.1040	0.6846	0.128*	
C13	0.5406 (4)	0.4958 (13)	0.6900 (3)	0.122	
H13A	0.4881	0.6034	0.6949	0.147*	
H13B	0.5656	0.5768	0.6555	0.147*	
C14	0.6167 (4)	0.4976 (13)	0.7569 (3)	0.123	
H14A	0.6367	0.6807	0.7676	0.184*	
H14B	0.6683	0.3891	0.7521	0.184*	
H14C	0.5912	0.4241	0.7913	0.184*	

### Atomic displacement parameters (Å^2^)

**Table:**

	*U*^11^	*U*^22^	*U*^33^	*U*^12^	*U*^13^	*U*^23^
N	0.055 (2)	0.056 (3)	0.086 (3)	0.014 (2)	0.008 (2)	−0.010 (2)
C1	0.088 (3)	0.040 (2)	0.050 (2)	−0.006 (2)	0.018 (2)	−0.0012 (19)
O1	0.079 (2)	0.073 (2)	0.122 (3)	0.031 (2)	0.016 (2)	0.010 (2)
C2	0.073 (3)	0.048 (3)	0.057 (2)	0.008 (2)	0.008 (2)	0.004 (2)
O2	0.063 (2)	0.111 (3)	0.138 (3)	0.010 (2)	0.040 (2)	0.022 (3)
C3	0.053 (2)	0.057 (3)	0.050 (2)	0.003 (2)	0.0052 (17)	−0.010 (2)
O3	0.0661 (19)	0.094 (3)	0.0691 (18)	−0.0031 (18)	0.0249 (15)	0.0283 (18)
O4	0.0533 (16)	0.074 (2)	0.0563 (16)	0.0033 (15)	0.0203 (13)	0.0139 (15)
C4	0.046 (2)	0.036 (2)	0.049 (2)	−0.0041 (17)	0.0126 (16)	0.0033 (18)
C5	0.051 (2)	0.044 (2)	0.051 (2)	−0.0027 (19)	0.0062 (18)	−0.0037 (18)
O5	0.075 (2)	0.070 (2)	0.0647 (19)	−0.0062 (17)	0.0305 (15)	0.0095 (17)
C6	0.057 (2)	0.065 (3)	0.0377 (18)	−0.003 (2)	0.0119 (18)	0.0054 (19)
O6	0.102 (3)	0.116 (3)	0.088 (2)	−0.025 (2)	0.042 (2)	0.016 (2)
C7	0.053 (2)	0.052 (3)	0.058 (2)	−0.019 (2)	0.010 (2)	−0.010 (2)
C8	0.048 (2)	0.071 (3)	0.083 (3)	−0.005 (2)	0.031 (2)	0.018 (2)
C9	0.053 (2)	0.077 (3)	0.078 (3)	0.010 (3)	0.032 (2)	0.021 (3)
C10	0.061 (3)	0.115 (4)	0.063 (3)	0.008 (3)	0.009 (2)	−0.011 (3)
C11	0.063 (3)	0.131 (5)	0.078 (3)	−0.010 (3)	0.018 (2)	0.002 (3)
C12	0.107	0.107	0.107	0.000	0.024	0.000
C13	0.122	0.122	0.122	0.000	0.028	0.000
C14	0.123	0.123	0.123	0.000	0.028	0.000

### Geometric parameters (Å, º)

**Table:**

N—O2	1.200 (4)	C7—H7A	0.9300
N—O1	1.216 (4)	C8—C9	1.476 (6)
N—C3	1.490 (5)	C8—C11	1.517 (6)
C1—C2	1.366 (5)	C8—H8A	0.9800
C1—C6	1.383 (5)	C10—H10A	0.9600
C1—H1A	0.9300	C10—H10B	0.9600
C2—C3	1.405 (5)	C10—H10C	0.9600
C2—H2A	0.9300	C11—C12	1.447 (6)
C3—C4	1.376 (5)	C11—H11A	0.9700
O3—C7	1.204 (4)	C11—H11B	0.9700
O4—C6	1.353 (4)	C12—C13	1.428 (7)
O4—C8	1.446 (4)	C12—H12A	0.9700
C4—C5	1.385 (5)	C12—H12B	0.9700
C4—H4A	0.9300	C13—C14	1.587 (7)
C5—C6	1.412 (5)	C13—H13A	0.9700
C5—C7	1.479 (5)	C13—H13B	0.9700
O5—C9	1.291 (5)	C14—H14A	0.9600
O5—C10	1.468 (4)	C14—H14B	0.9600
O6—C9	1.200 (5)	C14—H14C	0.9600
			
O2—N—O1	124.6 (4)	O6—C9—C8	121.4 (5)
O2—N—C3	116.9 (4)	O5—C9—C8	115.3 (4)
O1—N—C3	118.5 (4)	O5—C10—H10A	109.5
C2—C1—C6	121.6 (4)	O5—C10—H10B	109.5
C2—C1—H1A	119.2	H10A—C10—H10B	109.5
C6—C1—H1A	119.2	O5—C10—H10C	109.5
C1—C2—C3	117.6 (4)	H10A—C10—H10C	109.5
C1—C2—H2A	121.2	H10B—C10—H10C	109.5
C3—C2—H2A	121.2	C12—C11—C8	118.7 (5)
C4—C3—C2	123.2 (4)	C12—C11—H11A	107.6
C4—C3—N	119.5 (4)	C8—C11—H11A	107.6
C2—C3—N	117.3 (4)	C12—C11—H11B	107.6
C6—O4—C8	116.7 (3)	C8—C11—H11B	107.6
C3—C4—C5	117.8 (3)	H11A—C11—H11B	107.1
C3—C4—H4A	121.1	C13—C12—C11	113.7 (6)
C5—C4—H4A	121.1	C13—C12—H12A	108.8
C4—C5—C6	120.4 (4)	C11—C12—H12A	108.8
C4—C5—C7	117.3 (3)	C13—C12—H12B	108.8
C6—C5—C7	122.3 (4)	C11—C12—H12B	108.8
C9—O5—C10	117.2 (4)	H12A—C12—H12B	107.7
O4—C6—C1	125.9 (4)	C12—C13—C14	110.4 (6)
O4—C6—C5	114.7 (3)	C12—C13—H13A	109.6
C1—C6—C5	119.4 (4)	C14—C13—H13A	109.6
O3—C7—C5	123.5 (4)	C12—C13—H13B	109.6
O3—C7—H7A	118.3	C14—C13—H13B	109.6
C5—C7—H7A	118.3	H13A—C13—H13B	108.1
O4—C8—C9	113.1 (3)	C13—C14—H14A	109.5
O4—C8—C11	104.4 (3)	C13—C14—H14B	109.5
C9—C8—C11	110.5 (4)	H14A—C14—H14B	109.5
O4—C8—H8A	109.6	C13—C14—H14C	109.5
C9—C8—H8A	109.6	H14A—C14—H14C	109.5
C11—C8—H8A	109.6	H14B—C14—H14C	109.5
O6—C9—O5	123.2 (5)		
			
C6—C1—C2—C3	−1.3 (6)	C4—C5—C6—C1	1.5 (5)
C1—C2—C3—C4	1.0 (6)	C7—C5—C6—C1	−177.6 (3)
C1—C2—C3—N	−178.6 (3)	C4—C5—C7—O3	−1.5 (5)
O2—N—C3—C4	−10.1 (6)	C6—C5—C7—O3	177.6 (4)
O1—N—C3—C4	170.9 (4)	C6—O4—C8—C9	−72.8 (5)
O2—N—C3—C2	169.5 (4)	C6—O4—C8—C11	166.9 (4)
O1—N—C3—C2	−9.6 (5)	C10—O5—C9—O6	−3.7 (6)
C2—C3—C4—C5	0.6 (5)	C10—O5—C9—C8	−180.0 (3)
N—C3—C4—C5	−179.9 (3)	O4—C8—C9—O6	160.8 (4)
C3—C4—C5—C6	−1.8 (5)	C11—C8—C9—O6	−82.5 (6)
C3—C4—C5—C7	177.3 (3)	O4—C8—C9—O5	−22.8 (5)
C8—O4—C6—C1	4.5 (5)	C11—C8—C9—O5	93.8 (4)
C8—O4—C6—C5	−177.5 (3)	O4—C8—C11—C12	−57.5 (6)
C2—C1—C6—O4	178.0 (4)	C9—C8—C11—C12	−179.5 (5)
C2—C1—C6—C5	0.1 (6)	C8—C11—C12—C13	−68.5 (7)
C4—C5—C6—O4	−176.6 (3)	C11—C12—C13—C14	−157.4 (5)
C7—C5—C6—O4	4.3 (5)		

### Hydrogen-bond geometry (Å, º)

**Table:**

*D*—H···*A*	*D*—H	H···*A*	*D*···*A*	*D*—H···*A*
C12—H12*A*···O4	0.97	2.51	2.877 (6)	102
C7—H7*A*···O4	0.93	2.46	2.769 (5)	100
C2—H2*A*···O1^i^	0.93	2.52	3.442 (6)	169

Symmetry code: (i) −*x*, −*y*+2, −*z*+1.
